# Mapping cerebral pulse pressure and arterial compliance over the adult lifespan with optical imaging

**DOI:** 10.1371/journal.pone.0171305

**Published:** 2017-02-24

**Authors:** Chin Hong Tan, Kathy A. Low, Tania Kong, Mark A. Fletcher, Benjamin Zimmerman, Edward L. Maclin, Antonio M. Chiarelli, Gabriele Gratton, Monica Fabiani

**Affiliations:** 1 Department of Psychology, University of Illinois at Urbana-Champaign, Urbana, Illinois, United States of America; 2 Beckman Institute, University of Illinois at Urbana-Champaign, Urbana, Illinois, United States of America; University of Texas at Dallas, UNITED STATES

## Abstract

Cerebrovascular health is important for maintaining a high level of cognitive performance, not only in old age, but also throughout the lifespan. Recently, it was first demonstrated that diffuse optical imaging measures of pulse amplitude and arterial compliance can provide estimates of cerebral arterial health throughout the cortex, and were associated with age, estimated cardiorespiratory fitness (eCRF), neuroanatomy and cognitive function in older adults (aged 55–87). The current study replicates and extends the original findings using a broader age range (a new adult sample aged 18–75), longer recording periods (360 s), and a more extensive optical montage (1536 channels). These methodological improvements represent a 5-fold increase in recording time and a 4-fold increase in coverage compared to the initial study. Results show that reliability for both pulse amplitude and compliance measures across recording blocks was very high (*r*(45) = .99 and .75, respectively). Pulse amplitude and pulse pressure were shown to correlate with age across the broader age range. We also found correlations between arterial health and both cortical and subcortical gray matter volumes. Additionally, we replicated the correlations between arterial compliance and age, eCRF, global brain atrophy, and cognitive flexibility. New regional analyses revealed that higher performance on the operation span (OSPAN) working memory task was associated with greater *localized* arterial compliance in frontoparietal cortex, but not with *global* arterial compliance. Further, greater arterial compliance in frontoparietal regions was associated with younger age and higher eCRF. These associations were not present in the visual cortex. The current study not only replicates the initial one in a sample including a much wider age range, but also provides new evidence showing that frontoparietal regions may be especially vulnerable to vascular degeneration during brain aging, with potential functional consequences in cognition.

## Introduction

Many studies have demonstrated that vascular health plays an important role in both normal (pre-clinical) aging and in conditions that become more prevalent in aging, such as mild cognitive impairment (MCI) and Alzheimer’s disease (AD). Systemic arterial stiffening contributes to negative neurological outcomes, including poorer cognitive function [1–6], greater brain atrophy in multiple regions [7], increased risk of degenerative disease states such as AD [8–11] as well as increased cardiovascular mortality rates [12–13].

In comparison, vascular health in younger adults has received more limited attention [14], despite evidence showing that arterial aging begins early in life [15–16] and that the negative consequences of arterial stiffness in relatively young populations are associated with poorer white matter health and reduced gray matter volume [17]. These adverse outcomes are also compounded by other known cardiovascular risk factors, such as low cardiorespiratory fitness (CRF) and poor dietary intake [18–20], emphasizing the need for control and intervention strategies at younger ages [21–22] for the prevention of age-related cognitive decline.

Fabiani and colleagues [23] recently introduced a novel, non-invasive method to derive indices of cerebrovascular health using diffuse optical imaging. This work was based on a body of research in humans and animals demonstrating that optical measures are sensitive to vascular phenomena due to changes in near-infrared (NIR) light absorption during arterial blood pulsation, as a function of the cardiac cycle. In the periphery, information about heart rate and oxygen saturation can be extracted from photoplethysmographic waveforms recorded by pulse oximeters [24]. For example, recording from neonatal pig brains, Themelis and colleagues [25] found that near-infrared spectroscopic (NIRS) methods for quantifying cerebral blood flow hemodynamics were highly correlated with well-established laser-Doppler flowmetry methods [26]. In clinical application, Ebihara and colleagues [27] analyzed the pulse power spectrum using NIRS in patients with cerebral ischemia and found that pulse transmission in the ischemic cerebral hemisphere was smaller compared to the contralateral side, reflecting the reduced cerebral blood flow associated with ischemia.

Fabiani and colleagues [23] relied on diffuse optical methods and an extended recording montage to extract cerebral pulsatile waveforms from the adult human brain in a sample of middle age and older adults. From these waveforms, they quantified three indices reflective of different aspects of cerebrovascular health, namely, pulse amplitude, arterial compliance and pulse transit time. Pulse amplitude was conceptualized as a proxy measure of pulse pressure (systolic blood pressure minus diastolic blood pressure) in *cerebral* arteries, and this claim was supported by data showing that the two were highly correlated. Pulse amplitude measures were also positively correlated with age (as arterial stiffening induces an increase in the difference between systolic and diastolic pressure), but were not associated with eCRF, blood flow measured with arterial spin labeling (ASL; [28]), brain anatomy or cognitive function (as measured by neuropsychological tests).

Additional recent evidence from our laboratory supports the idea that optical pulse amplitude measures the temporary distension of the cerebral arteries caused by the movement of the pulse pressure wave across the vascular system. Tan and et al. [29] found that pulse amplitude measures can track both generalized and localized changes in cerebrovascular tone (vasodilation and vasoconstriction) as a function of voluntary breath-holding and a Sternberg memory task in a group of middle age and older adults. Further, estimates of cerebrovascular reactivity (CVR) derived from the pulse amplitude measures during the breath-holding task were also found to be negatively associated with age, and positively associated with performance on the modified mini-mental status examination (mMMSE; [30]). These data suggest that the utility of cerebral pulse amplitude goes beyond simply indexing pulse pressure in the brain, but also provides information about the cerebrovascular system that is related to aging and CVR.

Fabiani and colleagues [23] also found that arterial compliance, measured with diffuse optical methods, was negatively associated with age and positively associated with eCRF, brain volumes (i.e., overall preservation of white and gray matter volumes) and cognitive flexibility. In addition, regional pulse transit time, also measured with diffuse optical methods, was associated with performance on distinct cognitive tasks. Specifically, they found evidence for a double dissociation, whereby slower pulse transit time (indicating higher arterial elasticity) in the left middle cerebral artery (IMCA, feeding Broca’s area) was associated with higher performance on a verbal fluency task but not with performance in the operation span task (OSPAN, a test that provides an estimate of working memory capacity; [31]). The opposite relationship was found with pulse transit time in the superior portion of the precentral artery bilaterally (sPCA, feeding dorsolateral prefrontal cortex). The authors argued that this double-dissociation reflects the functional specialization of the cortical areas fed by these two arteries. They concluded that these indices of cerebrovascular health provide complementary information about the arterial system to that obtained with other approaches such as magnetic resonance imaging (MRI) and Doppler ultrasound.

Fabiani and colleagues [23] also highlighted some limitations and areas of possible future improvements. Specifically, they suggested that the relatively low spatial resolution of the method could be improved by using a denser array of optodes. In addition, the utility of extracting *regional* estimates of arterial compliance on cognition remained largely unknown, even though the finding that arterial compliance was associated with pulse transit time is suggestive of a possible link to cognition.

The purpose of the current study is therefore two-fold; first, we seek to replicate and extend the initial findings (which were obtained in a sample of older adults aged 55–87 years) to an evenly distributed sample spanning a much broader age range (18–75 years). The findings described in this paper thus represent the *first replication* (and considerable *extension*) of a study describing a brand-new way of assessing arterial health based on optical methods [23]. Given that the measurement approach is completely new, it is important to provide a replication on an *independent* sample, encompassing the age-range of the original sample but also extending it. This will allow us to investigate how *global* measures of cerebrovascular health relate to eCRF, global brain volumetric indices and cognitive function, not only in older adults but also over the adult life span starting in the late teens. Second, we increased the density of the optical recording montage from 384 to 1536 channels and increased recording time from 72 to 360 seconds, in order to increase the spatial resolution and reliability of the data. This allows us to not only replicate the previous findings but also to better explore relationships between *regional* measures of arterial compliance, age, and cognitive function.

Functional MRI studies have shown that both prefrontal and parietal regions are associated with performance on working memory tasks [32–37]. Other studies have shown that frontal and parietal regions are more prone to age-related volumetric losses than other regions such as the occipital cortex [38–39], that reductions in frontal and parietal lobe perfusion are associated with poorer cognitive performance [40–41] and that higher levels of CRF are associated with greater oxygenation in prefrontal regions [42] and more preserved prefrontal and parietal gray matter volumes [43–45].

Motivated by this fMRI- and sMRI-based evidence and by our initial findings that arterial compliance is associated with pulse transit time [23], here we investigated whether *regional* measures of arterial compliance in the frontoparietal cortex (Brodmann areas 9 and 7) would be more associated with performance on the OSPAN working memory task [31] compared to a *global* estimate of cerebral arterial compliance. Further, we also expected to find strong age-related reductions in arterial compliance in these regions but not in other regions such as visual cortex. Finally, we expected the association between eCRF and arterial compliance to be more evident for frontoparietal regions than for visual cortex.

Taken together, the results presented in this paper should provide support for the utility of these newly developed optical cerebrovascular indices of arterial health during the adult lifespan, show their reliability, and validate their predictive value with respect to indices of volumetric and cognitive health. Finally, they should also show the utility of deriving *regional* measures of cerebrovascular health in addition to global cerebral and systemic measures.

## Methods

By design, all screening and data collection procedures and parameters described in this Methods section are similar to those used in [23]. Participants, however, constitute a completely independent sample from those whose data are presented in [23].

### Participants

Forty-eight adults (age range = 18–75 years, mean age = 47.8, 25 females) were recruited through advertisements in local newspapers, campus-wide emails and postings at area gyms, retirement homes and community centers in the Urbana-Champaign community. In order to ensure an even spread across ages, the age range was divided into six decades (18–27, 28–37, 38–47, 48–57, 58–67, and 68–77), and 8 subjects were recruited for each decade. However, for the majority of analyses presented in this paper, age (in years) was used as a continuous variable. One subject (from an intermediate age group) had to be removed from all analyses involving pulse measures because we were unable to extract a suitable pulse trace from the optical data, possibly due to excessive movement, leaving a final sample count of 47 subjects. The demographic characteristics of the participants are summarized in Table 1. In this table, information is provided about the overall sample, as well as about three broad age groups (younger, middle-aged and older adults, each comprising two of the original decades used to generate the overall sample). This classification is provided to show that important variables such as years of education and IQ were consistent across the entire sample.

**Table 1:** Demographic characteristics of the participants.

Variable	All (N = 47)	Young (N = 16)	Middle (N = 15)	Old (N = 16)
	Mean (SD)	Mean (SD)	Mean (SD)	Mean (SD)
Age (years)	47.6 (17.5)	27.2 (6.1)	48.8 (6.2)	66.9 (5.0)
Education (years)	17.3 (2.2)	16.4 (2.3)	16.9 (1.8)	18.5 (2.2)
mMMSE	55.8 (1.2)	55.4 (1.2)	56.1 (1.1)	55.8 (1.3)
Shipley’s Vocabulary Test	34.7 (3.7)	33.4 (2.7)	33.5 (4.1)	37.1 (3.0)
Beck’s Depression Index	2.2 (2.5)	2.6 (2.2)	3.0 (3.0)	1.2 (2.1)
K-BIT2 (IQ)	116.4 (10.2)	116.3 (7.8)	113.1 (12.0)	119.6 (10.2)
Raven’s Progressive Matrices	9.7 (3.0)	12.1 (2.0)	9.5 (2.5)	7.5 (2.6)

### Screening procedures

Prospective participants were screened based on a series of health and cognitive criteria. Subjects who had serious or chronic medical conditions or a history of major neurological and psychiatric disease or drug abuse were excluded from participation. In addition, subjects with a score of less than 51 on the mMMSE [30], more than 15 on the Beck’s Depression Inventory [46] or who reported smoking more than half a pack of cigarettes and/or consuming in excess of two alcoholic drinks per day were also excluded. Three participants reported taking blood pressure medications (diuretics) and 2 others reported taking statins due to high cholesterol, which may indirectly affect blood pressure. All participants were right-handed (as assessed by the Edinburgh Handedness Inventory [47]), had normal or corrected-to-normal vision, and were native speakers of English. The Institutional Review Board of the University of Illinois at Urbana-Champaign approved all aspects of the study. Prior to participation, all participants signed informed consent documents.

### Assessment of cognitive function

A battery of neuropsychological tests was given to all participants, and consisted of the following: the Kaufmann Brief Intelligence Test Second Edition (K-BIT2 [48]) and the Raven’s progressive matrices [49] to estimate crystallized and fluid IQ, the vocabulary sub-test of the Shipley-Institute of Living Scale [50] to measure vocabulary, the Wisconsin Card Sorting Test (WCST [51–52]), the Controlled Oral Word Association sub-test of the Multilingual Aphasia Examination (a measure of verbal fluency using the letters CFL [53]), the OSPAN task [32], the Trail Making Tests A and B [54], to measure working memory and executive function, and the Logical Memory I and II tasks from the Wechsler Memory Scale–Fourth Edition (WMS–IV [55]) to measure episodic memory. Scores for forward and backward digit span were derived from the mMMSE.

### Assessment of cardiorespiratory fitness

We estimated CRF with an equation that utilizes easily acquired parameters that are highly predictive of VO_2max_ (*r* ≈ .7 [56–57]). This measure is derived by using a linear combination of weighted variables (gender, age, body mass index [BMI], resting heart rate, a physical activity score and a numerical constant). This eCRF index, expressed in metabolic equivalents, was first developed in a large sample of men and women (N > 40,000, 20–70 years of age), which corresponds well to the age range of our participants. This CRF estimate has also been validated in samples of older adults [58–59]. In this study, we partialed out gender from analyses involving eCRF to control for systematic differences in CRF between males and females, which are known to be present when using VO_2max_ to assess fitness [56], and would not be expected to reflect real variations in fitness or vascular health.

### Experimental procedures and types of measures

The data presented here were collected as a part of a much larger, multi-session project intended to investigate brain function using diffuse optical and magnetic resonance imaging (MRI) methods. Session 1 involved neuropsychological assessments and familiarization to MRI scanning within a mock magnet. Session 2 involved the collection of structural MRI data (used for the anatomical co-registration of optical data and for brain volume estimations). Session 3 included collection of optical imaging data from visual cortex only (not included in this report). In session 4, we collected the optical imaging data and lead I of the electrocardiogram (EKG) for time-locking the arterial pulse. These are the data presented here. Participants’ blood pressure was taken during sessions 2–4 and averaged to provide more stable estimates of their systolic and diastolic blood pressure.

### Electrocardiogram recording and analysis

Lead I of the EKG (left wrist referenced to right wrist, impedance below 20 kOhm) was recorded with a Brain-Vision™ recorder and a Brain-Vision professional BrainAmp™ integrated amplifier system (Brain Products GmbH, Germany) with a sampling rate of 1000 Hz. The EKG data were extracted using EEGLab [60] and the optical pulse data were time-locked to the R-wave of the EKG. Each R-wave peak was identified using an algorithm running on MATLAB R2014b (MathWorks, Natick, MA) that implemented a band-pass filter of 0.5–40 Hz, searched for peaks exceeding a standardized voltage threshold, and discarded points that fell outside the normal range of interbeat intervals. In less than 10% of subjects, this threshold had to be adjusted to ensure accurate R-wave detection. Manual visual inspection was also performed on each participant’s data to ensure that any misidentifications of R-wave peak were discarded.

### MRI acquisition and processing

Structural magnetic resonance images (sMRI) were collected for each participant using a 3T Siemens Trio full body scanner. A high resolution, 3D MPRAGE protocol was used, with flip angle = 9°, TE = 2.32 ms, TR = 1900 ms, and inversion time = 900 ms. MR slices were obtained in the sagittal plane (192 slices, .9 mm slice thickness, voxel size .9 x .9 x .9 mm) having matrix dimensions of 192 x 256 x 256 (in-plane interpolated at acquisition to 192 x 512 x 512) and field of view of 172.8 x 230 x 230 mm. All images were visually examined by multiple researchers and no significant defects or distortions were discovered. Cortical reconstruction and volumetric segmentation were performed with the FreeSurfer 5.3 image analysis suite (http://surfer.nmr.mgh.harvard.edu/; e.g., [61–65]. FreeSurfer morphometric procedures have been demonstrated to show good test-retest reliability across scanner manufacturers and across field strengths [65]. These procedures are similar to what was in used in [23] but with a newer version of Freesurfer.

### Optical recording and analysis

#### Recording

**Fig 1:**
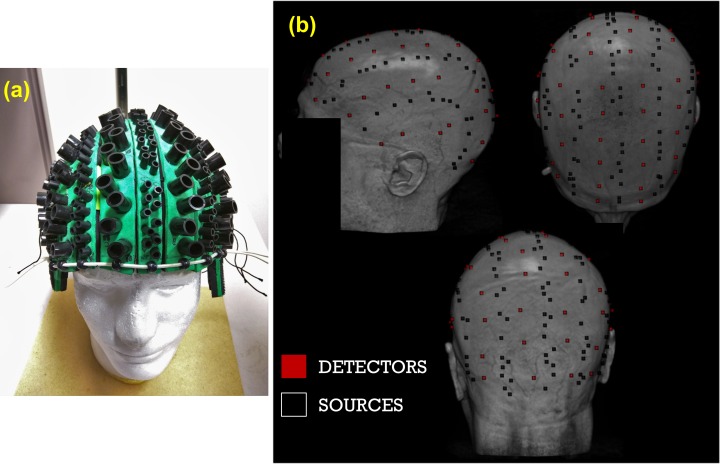
(a) Photograph of the soft foam, adjustable optical recording cap used in the study. Detectors were fitted into the larger tubes while sources were fitted into the smaller tubes. The cap was further secured to the subject’s head by tightening an elastic band around its circumference of the cap and through the usage of a custom-built chin strap. (b) Locations of sources (black) and detectors (red) digitized in one representative participant, and plotted over the corresponding surface-rendered structural MRI (left, top and back views).

Optical data were recorded with six integrated frequency domain oxymeters (Imagent; ISS Inc., Champaign, IL). Data were collected from 24 detectors, each measuring light emitted by 16 time-multiplexed sources (384 channels), arranged in 4 different optical montages designed to cover the majority of the head. Sources and detectors were held flush to the participants’ scalp using a custom-built, soft foam, adjustable cap (Fig 1A), giving rise to a total of 1536 channels (i.e., source-detector pairings; Fig 1B). This represents a four-fold increase in channels compared to what was used by Fabiani et al. [23]. Laser diodes generated light at 830 and 690 nm (max amplitude: 10 mW, mean amplitude after multiplexing: 1 mW), modulated at 110 MHz. The light from the diodes was transmitted to the surface of the head by optic fibers (diameter = 400 μm; one fiber per emitter, with separate fibers carrying light at each of the two wavelengths, coupled at each location). Light was collected from the head using detector fiber bundles (diameter = 3 mm) connected to photomultiplier tubes (PMTs) fed with a current modulated at 110.003125 MHz, generating a 3.125 kHz cross-correlation frequency. A Fast Fourier Transform of the PMT output data was used to calculate DC (average) intensity, AC (amplitude), and relative phase, or photon, delay (in picoseconds). The analyses reported here are based on the AC intensity values. Optical parameters were sampled at 39.0625 Hz (25.6 ms per sampling point). The arterial pulse is one of the largest phenomena that can be measured using AC intensity and, being sensitive to arterial blood, originates almost entirely from oxy-hemoglobin, being easily visible on single trials [23]. The associations reported here are therefore driven by oxy-hemoglobin and not deoxy-hemoglobin concentration.

#### Co-registration with structural MRI

The spatial locations of individual optical sources and detectors were digitized using a Polhemus “3Space” FASTRAK 3D digitizer (Polhemus, Colchester, VT) while using the location of the nasion and preauricular locations as reference points. T1-weighted structural magnetic resonance images were acquired for every subject. The Polhemus digitization points were then co-registered with the MR images first using the three fiducial markers and then surface fitting the entire set of digitized points to the estimated scalp surface based on a Levenberg-Marquardt algorithm (least-squares fit) using in-house software (Optical Co-registration Package, OCP [66]). Co-registration using this procedure has been shown to result in errors of less than 4 mm [66–67]. Fig 1B shows the digitized recording montage of one representative participant superimposed on the corresponding structural MRI.

#### Measurement of pulse parameters

**Fig 2:**
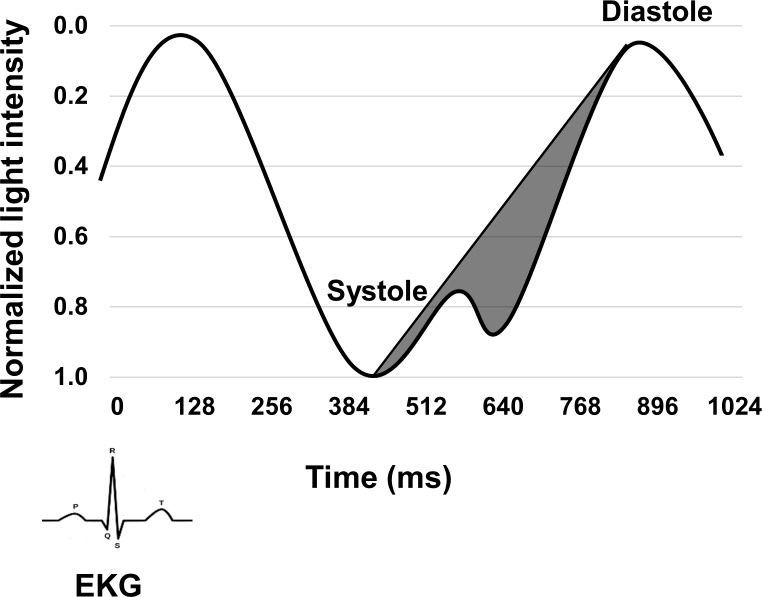
Representative cerebral pulse waveform of a single subject, normalized for both time (i.e., the interval between the systolic and diastolic peaks) and amplitude (with 1 being the systolic peak and 0 being the diastolic peak). The gray area corresponds to the estimated arterial compliance for the subject.

Measurements were taken at rest for 8 blocks with each block lasting 6 minutes. Each of the 4 montages was recorded for a total of 2 blocks (12 minutes total recording time for each montage), with the sequence of 4 montages counterbalanced across subjects within each age sextile. The optical data were then normalized and a band-pass filter between 0.5-5Hz was applied. Pulse waveforms were then extracted from each channel by averaging AC light intensity, time-locked to the peak of the EKG R wave. Pulse epochs occurring during all blocks were then averaged together and baselined to the first peak diastole period occurring between 128–256 ms. In-house software, “Opt-3d” [68], was used to combine channels whose mean diffusion paths (modeled as a curved ellipsoid) intersected for a given brain volume [69]. Only source-detector distances between 2 and 6 cm were used in the analysis. Similar to Fabiani et al. [23], pulse amplitude was defined as the mean AC amplitude in an interval between 384 and 538 ms after R wave onset, an interval during which the peak systolic phase occurred in all subjects and for all brain regions. The arterial compliance measure was computed by calculating the area under the pulse waveform between the peak systole and the peak diastole, normalized by both time (i.e., the interval between the systolic and diastolic peaks) and amplitude (with 1 being the systolic peak and 0 being the diastolic peak) and subtracted by a constant value of 0.5 (see Fig 2). This constant value was subtracted in order to compare the area of the pulse wave measured, with a triangular area representing a hypothetical linear decrease of the pulse amplitude after the systolic peak. Both pulse amplitude and arterial compliance data were extracted from an ROI comprising voxels covering most of the axial cortical surface (Talairach coordinates; X-axis: -45 to 45, Y-axis: -80 to 50). The signal-to-noise ratio (SNR) of near-infrared (NIR) pulse measurements is intrinsically high (approximately 2:1), as individual heart beats are clearly identifiable in a single optical channel. Due to filtering and averaging across multiple pulsations (>100), a substantially higher SNR was obtained in this study (reaching SNR levels of >20:1 at the single channel level). Regarding image reconstruction procedures, imaging the optical pulse shares identical procedures to the those developed for imaging slow hemodynamic oxygen fluctuations in the brain using Diffuse Optical Tomography (DOT), thus providing the same advantages and limitations [70]. Increasing channel coverage (field of view) and channel density provides more precise image reconstruction and higher image SNR in DOT. In addition, the denser optical array available in this study provides a higher SNR, with a two-point spatial resolution (i.e., the minimum distance between two perturbations that can be separated by the image reconstruction procedure) of <24 mm up to a depth of 30 mm [70]. It is now possible to compute arterial compliance on pulse waveforms in individual voxels and then average them, as opposed to computing it on a single waveform averaged over the axial surface (as it was done by [23]).

The global measurements of pulse amplitude and arterial compliance obtained in this manner were used for all analyses except when investigating regional effects of arterial compliance on OSPAN, where we extracted arterial compliance measurements from ROIs based on Brodmann area boundaries (as defined by [71–72]). For the reliability analyses, pulse amplitude and arterial compliance measures were extracted and compared between the block 1 and block 2 data that were collected for each of the 4 montages. For all other analyses, blocks were collapsed together.

### General analytic approach

The analyses presented in this paper are designed to examine changes in cerebral pulse amplitude and arterial compliance as a function of age, first at the global level and then in some selected regions (regional analysis). Age was treated as a continuous variable for all statistical analyses. However, to create figures facilitating comparisons across age groups, and for mapping age-related differences in pulse parameters on the brain, subjects were divided into three age groups (as described in Table 1). Given that the goals of this paper were to replicate and extend the findings reported by Fabiani et al. [23] and to investigate known brain regions involved in the OSPAN task, one-tailed tests were used based on our a-priori directional hypotheses. The data used in the analyses are available in S1 Data.

## Results

### Effects of age and eCRF

Here we summarize data indicating that our sample exhibits the typical relationships between age, eCRF, cognitive function and neuroanatomy. As expected, age was negatively correlated with eCRF after partialing out gender, *r*(44) = -.74, *p* < .001. Controlling for estimated total intracranial volume (eTIV), age also showed significant negative correlations with global brain volumetric measures, cortical white matter (*r*(44) = -.43, *p* = .002), cortical gray matter (*r*(44) = -.82, *p* < .001) and subcortical gray matter (*r*(44) = -.76, *p* < .001).

Correlations with eCRF were similar, with higher eCRF being associated with greater cortical white matter (*r*(43) = .29, *p =* .026), cortical gray matter (*r*(43) = .63, *p <* .001) and subcortical gray matter (*r*(43) = .56, *p <* .001), controlling for both gender and eTIV. These results are in line (with respect to both magnitude and direction) with those reported by Fabiani et al. [23] and others (see [73] for a review). These results are also in agreement with findings showing that higher eCRF is associated with reduced atrophy in brain volumetric measures (e.g., [44–45], [74]; see [75] for a discussion of the overlap between the effects of age and fitness on regional brain anatomy).

### Reliability of pulse parameters

**Fig 3:**
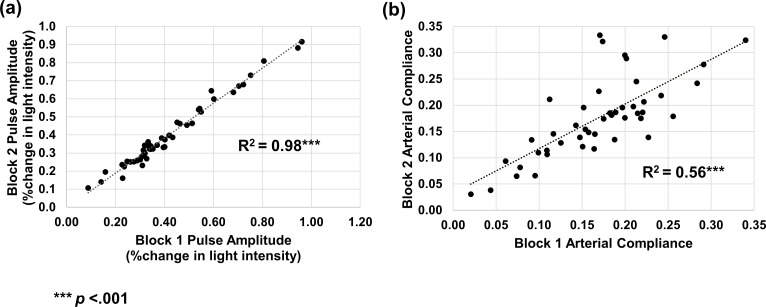
(a) Correlation between block 1 and 2 for pulse amplitude (expressed in percent change in light intensity). (b) Correlation between block 1 and 2 for arterial compliance (compliance is a dimensionless measure indicating the change of the shape of the diastolic section of the pulse from a straight line, with 0 indicating a straight, oblique line, and 0.5 indicating a flat line lasting the whole duration of the diastole followed by a vertical ascent).

We first set out to assess the reliability of pulse amplitude and arterial compliance measures by comparing the measures obtained in blocks 1 and 2, extracted from all 4 montages. Pulse amplitude measurements were highly reliable from block 1 to block 2, *r*(45) = .99, *p* < .001 (Fig 3A). The relative wide range seen in the pulse amplitude measure may be in part due to the broad age range encompassed by the current sample. Another contributing factor may be variability in light penetration across individuals and ages, which may affect the depth at which measures are taken. Data from one additional subject were removed from the arterial compliance reliability analyses because they were statistical outliers, Z score > |2.5|. Arterial compliance was also highly reliable across blocks, *r*(44) = .69, *p* < .001 (see Fig 3B). This replicates our initial findings [23] that both measures show good reliability, with pulse amplitude being more stable than arterial compliance. The reliability of arterial compliance was higher in the current study, *r*(44) = .75 compared to our previous report, *r*(51) = .56, which may be due to a number of factors, including (a) the use of a denser optical array; (b) the increase in recording time from 72 to 360 s; and (3) the computation of arterial compliance on waveforms at the voxel level before averaging. For all other analyses, data from all blocks were combined.

### Relationship between cerebral pulse amplitude and other variables

**Fig 4:**
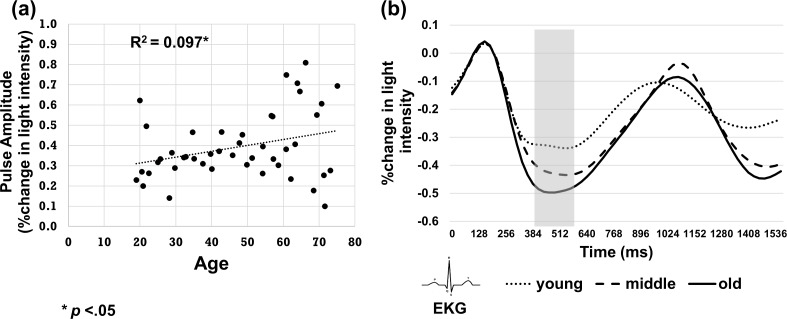
(a) Scatter plot showing that older age is associated with larger pulse amplitude (expressed as percent change in light intensity). (b) Average pulse waveforms split into age tertiles. The transparent gray bar indicates the time interval in which pulse amplitude values were extracted and averaged.

Of the 47 subjects, two were removed from the pulse amplitude analysis as they were outliers on the pulse amplitude measure (Z score > |2.5|) after combining data from all blocks together. Cerebral pulse amplitude was found to be positively correlated with pulse pressure measured at the arm (*r*(43) = .36, *p =* .008). As seen in Fig 4A, pulse amplitude was also significantly correlated with age, *r*(43) = .31, *p* = .019). This figure suggests the presence of increased pulse amplitude variability with age. In order to test whether this was indeed the case, we first computed squared standardized residuals after regressing pulse amplitude on age to determine each subject’s deviation in pulse amplitude from the predicted mean of their age group. A correlation of these residuals with age confirmed that older age was associated with greater variance in pulse amplitude (*r*(43) = .42, *p* = .002). This method of analysis allowed us to investigate variations in pulse amplitude as a continuous variable, as opposed to artificially dichotomizing subjects into young and old, which could result in a loss of power or in misleading results [76]. Increased variation of pulse amplitude with age may be due to the increase in pulse pressure typically found in older adults, which in turn increases the range (and therefore the variation) of possible values in pulse pressure and consequently, in pulse amplitude (which is highly correlated with pulse pressure). In a few subjects, variations in pulse amplitude may also reflect the influence of blood pressure medications.

We further investigated whether this increase in variation with age was mediated by eCRF by using PROCESS model 4 macro in SPSS, based on a series of ordinary least square (OLS) regression models [77]. After regressing age and gender from eCRF for the analysis, age was entered as the predictor variable, variation in pulse amplitude as the criterion variable and eCRF as the mediator. This mediation analysis, based on 5000 bootstrap samples, did not reveal a significant mediation effect of eCRF on the association between age and variation in pulse amplitude, 95% CI [-.0579, .0180]. Gold standard quantification of cardiorespiratory fitness using VO_2max_ measures may allow us to better explain the greater variation in pulse amplitude for older subjects in the future. This may also reduce the correlations between pulse amplitude measures and other variables in the experiment. Fig 4B shows the cerebral pulse waveforms after dividing subjects into three age groups (see Table 1). The waveforms show a graded increase in pulse amplitude with age.

After controlling for gender, higher eCRF was not associated with lower pulse amplitude (*r*(42) = -.24, *p* = .056). Although this relationship was also not found by Fabiani et al. [23], the effect size in the current study was stronger in the predicted direction (*r*(42) = -.24 *p* > .05 compared to *r*(50) = .083, *p* > .05), which suggests that this association may be small but could become significant with greater sample size. Similar to Fabiani et al. [23], higher pulse amplitude was not associated with lower cortical white matter volume (*r*(42) = -.21, *p* = .091). However, we did find that higher pulse amplitude was associated with lower cortical gray matter volume (*r*(42) = -.33, *p* = .015) and subcortical gray matter volume (*r*(42) = -.28, *p* = .035), controlling for eTIV. Similar to what was reported by Fabiani et al. [23], smaller pulse amplitude was not correlated with better performance on any neuropsychological tests.

### Relationship between cerebral arterial compliance and other variables

**Fig 5:**
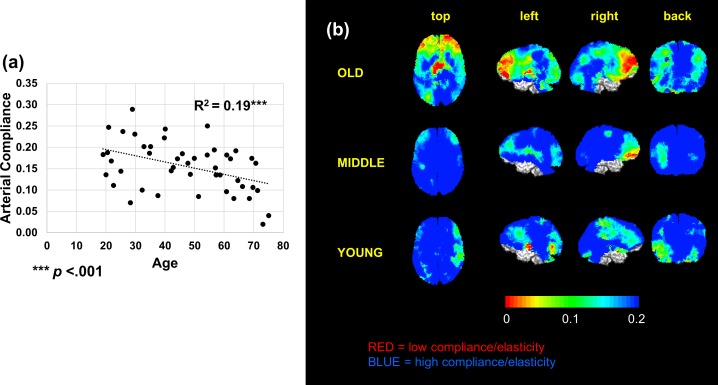
(a) Older age is associated with lower arterial compliance (i.e., greater arterial stiffness). b) Arterial compliance maps derived from age tertiles. Cortical arterial compliance is projected onto the brain surface. Compared to younger subjects, older subjects appear to show poorer arterial compliance (red regions), especially in prefrontal areas.

Out of the sample of 47 subjects, one was removed from the arterial compliance analysis due to being a statistical outlier, Z score > |2.5|. Unlike pulse amplitude, arterial compliance was not associated with pulse pressure, *r*(45) = 0.032, *p* >.05. Fig 5A shows that cerebral arterial compliance declines with age (*r*(44) = -.43, *p* < .001), replicating and extending previous findings. In addition, regional variations in arterial compliance are shown in Fig 5B for each of the three age groups. On average, arterial compliance decreased at a rate of 0.8% per year within this age range. It must be noted, however, that due to limitations in NIR penetration, the measurement of arterial compliance is derived largely from superficial projections of cortical data in the gray matter, and does not extend into deep white matter and subcortical regions. Unlike the pulse amplitude parameter, the variability in arterial compliance did not increase with age (*r*(44) = -.11, *p* = .24). This may be because those factors most likely to lead to an increase in pulse amplitude variability with age (i.e., the fact that the amplitude signal is large and that light penetration increases with age) do not directly affect the compliance estimates. In fact, compliance measures decrease with age and penetration is not a confound, since the measure of compliance only refers to the shape, and not the amplitude, of the pulse signal. Greater eCRF was associated with higher arterial compliance (*r*(43) = .32, *p* = .017), controlling for gender.

**Fig 6:**
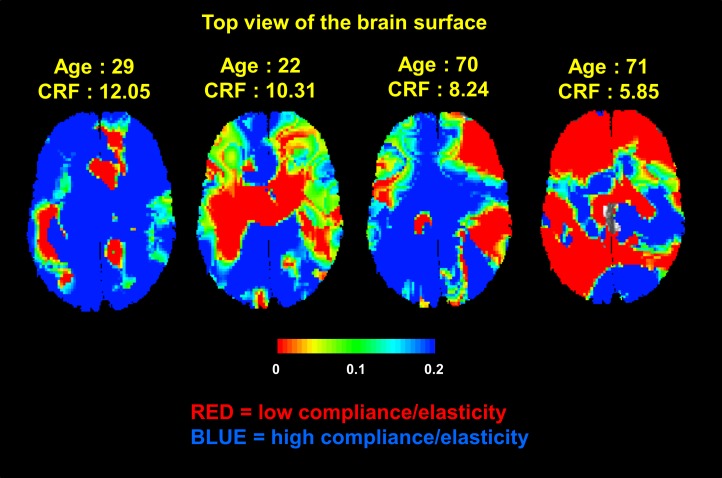
Maps of cortical arterial compliance estimates in four representative subjects varying in age and eCRF. In each of the maps cortical arterial compliance is projected onto the brain surface (top view). In general, subjects who are younger and relatively more fit show greater arterial compliance (blue regions).

Fig 6 shows compliance maps (derived from cortical projection of optical data) for 2 younger and 2 older representative subjects, varying in fitness levels. In general, subjects who are younger and relatively fitter within their age group have higher arterial compliance. Better arterial compliance was also positively correlated with cortical white matter volume (*r*(43) = .29, *p* = .028), cortical gray matter (*r*(43) = .35, *p* = .010) and subcortical gray matter (*r*(43) = .37, *p* = .007), controlling for eTIV. Similar to data presented in Fabiani et al. [23], arterial compliance was also not correlated with pulse amplitude, (*r*(42) = -.047, *p* = .76), suggesting again that these are separate indices of brain arterial function.

Two subjects did not complete the WCST. Higher global arterial compliance was associated with many components of the WCST, including total number of trials (*r*(42) = .27, *p* = .037), number of errors (*r*(42) = −.33, *p* = .014), number of perseverative responses (*r*(42) = −.28, *p* = .032), number of perseverative errors (*r*(42) = −.31, *p* = .021) and number of non-perseverative errors (*r*(42) = −.32, *p* = .016), replicating previous findings that indicate that lower arterial compliance is associated with poorer cognitive flexibility.

### Relationship between regional arterial compliance and other variables

**Fig 7:**
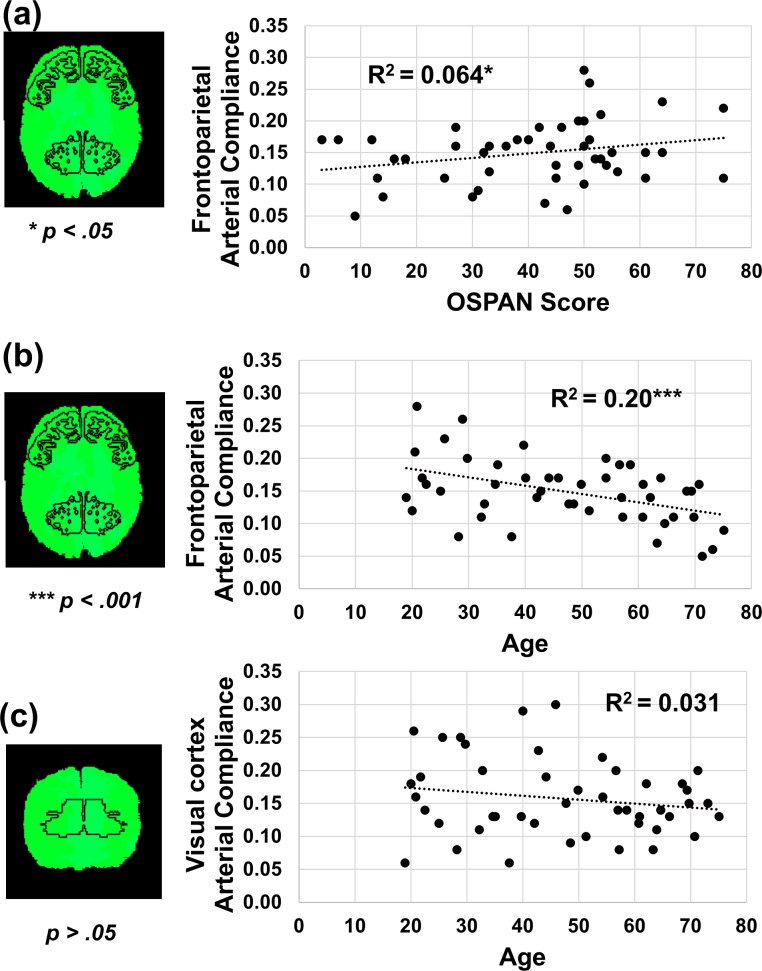
(a) Greater regional arterial compliance in frontoparietal regions (BA7 and BA9) was associated with performance on the OSPAN task. (b) Lower regional arterial compliance in frontoparietal regions (BA7 and BA9) was also associated with older age. c) Arterial compliance in the visual cortex (BA17 and BA18) was not associated with age.

One of the main advantages of these new optical methods is their potential to investigate not only the global effects of changes in cerebrovascular health, but also *regional*, localized effects that are not easily investigated with other means. Here we focused on specific regions that are known to support working memory function (for a review see [78]) and hypothesized that greater arterial compliance in these regions would be predictive of better performance in the OSPAN task. We extracted arterial compliance measures from frontoparietal regions (BA 9 and BA 7) and averaged them, and found the predicted association (*r*(44) = .25, *p* = .045, see Fig 7A). This relationship was not found when correlating OSPAN performance with global arterial compliance (*r*(44) = .18, *p* = .11). These data therefore provide evidence suggesting that there is added value in extracting regional measures of arterial compliance, permitting to examine relationships that might be washed out when global measures of arterial compliance are used. Further, as hypothesized, we found that arterial compliance in frontoparietal regions (BA 9 and BA 7) decreased with age (*r*(44) = -.45, *p* < .001) and increased with eCRF (*r*(43) = .38, *p* = .004) while arterial compliance in the visual cortex (BA 17 and BA 18) was not associated with age (*r*(44) = -.17, *p* = .12) or eCRF (*r*(43) = .13, *p* = .19), controlling for gender (Fig 7B and 7C). Taken together these results suggest that, similar to regional variations in age-related brain atrophy [38–39], hypoperfusion [40–41] and the neuroprotective benefits of fitness (see [44] for a review), reductions in arterial compliance in the brain also do not occur at similar rates in all regions, and that regional reductions in arterial compliance may have differential impact on cognition depending on where it occurs.

## Discussion

The results presented in the current paper represent evidence supporting the novel use of diffuse optical imaging methods to study cerebrovascular health and its functional consequences on cognitive decline over the course of life span development and aging. The importance of these findings is underscored by growing evidence showing that disruptions in the microcirculatory processes in the brain’s vasculature are closely linked to neurodegeneration in cognitive and brain aging [79–80]. In addition, a large scale study involving over 6000 autopsy reports found that patients with AD had a significantly higher association with cerebrovascular disease and vascular pathology than other neurodegenerative diseases, suggesting that targeting cerebrovascular pathology for early intervention may help prevent or slow down the clinical manifestations of AD [81]. This idea is in line with evidence showing that apolipoprotein E genotype and cerebrovascular pathology such as poor CVR and carotid artery intima-media thickness (IMT) can increase the risk of conversion from mild cognitive impairment (MCI) to AD [82]. Given that the optical pulse amplitude measure can be used to quantify CVR by inducing hypercapnia [29] and arterial compliance acts as an index of vascular stiffness, these measures may also be useful in predicting conversion from MCI to AD. Within this larger context, the results from the current study support the critical role of cerebrovascular health in brain and cognitive decline in the normal aging process. We have largely replicated the initial findings reported by Fabiani et al. [23] in a sample including only older adults, and extended them to a wider age range including much of the adult lifespan (18–75).

For pulse amplitude, we replicated the positive correlations with age and pulse pressure reported by Fabiani and colleagues [23]. We did not find any pulse amplitude associations with cortical white matter volume, eCRF and cognitive function. However, we found that larger pulse amplitude was associated with reduced cortical and subcortical gray matter volumes. This new evidence of associations between pulse amplitude and gray matter volume may be in part driven by the increase in signal-to-noise ratio (SNR) and the wider age-range available in the current study. These results are consistent with our original interpretation that, similarly to systemic pulse pressure measurements, pulse amplitude measures reflect both the long-term properties of the cerebrovascular system and also other factors such as the current state of the cerebrovascular tone (see also [29]).

For arterial compliance, we replicated our initial findings that greater arterial compliance is associated with younger age and higher eCRF, is predictive of global white and gray matter volume, as well as cognitive flexibility (as measured by the WCST). Many of the correlations between pulse amplitude and arterial compliance with age, eCRF, anatomy and cognitive function were very similar across both studies. These results suggest that we can reliably measure pulse amplitude and arterial compliance both within and across studies. Maps of arterial compliance in the three age groups also showed clear differences, with older adults showing less compliance, especially in prefrontal regions. We empirically tested the functional consequences of the differences shown by the arterial compliance maps and found that arterial compliance in frontoparietal regions was associated with working memory performance indexed by the OSPAN task. These specific regional findings (which are reported here for the first time) thus suggest that, although global measures of arterial compliance can provide a quick overview of the general state of cerebral arterial health, regional arterial compliance may be more useful for investigating age- and fitness-related declines in specific cognitive domains. These new results can motivate future studies aimed at investigating a wide range of task-specific effects of regional arterial compliance.

Other studies have used methodologies such as transcranial Doppler (TCD) ultrasound (see [83] for a review) and ASL [84–85] to investigate the relationship between cerebrovascular health and cognition. However, these measures lack the level of sensitivity to regional specificity afforded by diffuse optical imaging. TCD ultrasound relies on measuring blood flow velocity as an index of cerebral arterial compliance but is limited to the insonation of a few large arteries such as the middle cerebral artery (MCA). Likewise, although absolute measurements of cerebral blood flow using ASL can be derived, they are still limited by their intrinsically low SNR [86], which restricts ASL utility for examining regional relationships with domain-specific cognitive function associated with the OPSAN task and others. Our novel regional findings thus represent not only an advancement over previous work [23], but also demonstrate the advantages involved in using diffuse optical imaging over other modalities.

The regional associations of arterial compliance and working memory performance index by the OSPAN task may provide an explanation for ceiling effects seen in blood-oxygen-level dependent (BOLD) functional MRI studies on working memory. Schneider-Garces et al. [34] suggested that ceiling effects seen in both regional BOLD activity and performance at high memory loads may be due to either a limitation in working memory capacity (“cognitive” explanation) or a limitation in vascular supply (“energetic explanation”). Variation in regional compliance may provide some support for the “energetic explanation”. In other words, changes in regional neuronal activity as a function of cognitive load and its subsequent hemodynamic consequences may be both mediated by the physiological compliance of the corresponding regional arterial supply. Future studies using concurrent fMRI and diffusive optical imaging may be able to further test this hypothesis.

Arterial compliance was also not associated with pulse amplitude and pulse pressure. Chronically high blood pressure has been associated with arterial wall damage, resulting in loss of elasticity (and therefore of compliance) of the arterial wall [87–89]. This may lead to the prediction of a correlation between blood pressure and arterial compliance, a prediction that is not supported by our findings. In fact, in our data, it is the compliance measure (and not the peak-to-peak optical pulse amplitude measure related to blood pressure) that is a good predictor of brain volumetric changes and age-related cognitive decline. This apparent contradiction may depend on two factors. First, measures of blood pressure taken at the time of the experiment do not necessarily reflect the *history* of blood pressure across the lifetime, but only represent a time point that can be influenced by a number of other factors, such as, for instance, the level of anxiety at the time of measurement. Second, factors other than blood pressure (such as LDL cholesterol level and/or genetic predispositions) may contribute to the build-up of plaques resulting in the narrowing of arteries and interference with normal blood flow [90], as well as arterial wall damage, loss of myogenic tone and remodeling of the arterial wall [91]. All these phenomena, including chronically high-blood pressure, exert their effects on arterial wall elasticity (and on the resulting compliance) over an extended period of time [92]. There is therefore a very important added value in measuring directly arterial elasticity in the brain, rather than relying only on peripheral blood pressure measures, to assess the state of brain arteries. This is also consistent with data showing that systemic arterial compliance quantified using PWV measures is an independent predictor of vascular pathology and provides prognostic value over pulse pressure alone [93]. Cruickshank et al. [94] suggests that poor aortic arterial compliance reflects an integrated index of vascular damage over time, resulting in generalized “wear and tear” from repeated distension and recoil of blood vessels, and that multiple extraneous factors also contribute, such as smoking, lipidic dysfunction and glucose metabolism.

These results align with studies showing not only that arterial aging occurs early in life [15], but also that low physical activity in adolescents and young adults is independently associated with poorer arterial compliance [95]. In combination with studies showing that even modest increases in moderate-to-vigorous physical activity can improve systemic arterial stiffness measured using brachial-ankle pulse wave velocity (baPWV) in young adults [96], these studies provide converging evidence that although vascular risk factors accumulate from a young age, they are also amenable to change with adequate increase in physical activity. Although our results are consistent with these interpretations, whether increases in physical activity level can affect not only systemic arterial stiffness but also regional cerebral vascular stiffness, and whether it results in any improvement in cognitive function remain to be seen. Future studies should focus on using longitudinal intervention designs to elucidate these relationships. The current study also points to the utility of using both pulse amplitude and arterial compliance measures to investigate associations with the incidence of stroke. Given that cerebral small vessel disease (SVD) is known to be associated with greater risk of strokes [97], reducing pulse pressure and improving arterial compliance may be protective against cerebral infarcts. In cases of traumatic brain injury (TBI), pulse amplitude measures may also be useful for monitoring secondary injury (neurological damage after initial impact) that is associated with greater intracranial pressure followed by reduction in cerebral perfusion [98].

Limitations of the current study include the limited penetration of diffuse optical imaging, which precludes the examination of arteries that flow through white matter and subcortical regions. Therefore, inferences about the state of cerebrovascular health beyond cortical and superficial white matter brain regions should be made with caution, especially in adults. Future studies involving the collection of arteriograms to mask cerebral arteries at varying depths may allow us to compare pulse amplitude and arterial compliance in gray versus superficial white matter. A second limitation is the relatively low spatial resolution of the method. We have addressed the issue of low spatial resolution by using a much denser optical array in the current study, but this comes at the cost of an extended optical recording session which may limit its use in clinical settings. However, given that the associations between the pulse parameters and other important variables are very similar in both the initial study and the current study, it may be the case that a relatively sparse recording array is sufficient to see these effects.

In summary, the results of the current study replicated and extended our initial findings that noninvasive measures derived from diffuse optical imaging can index cerebrovascular health. This is also the first study demonstrating the utility of regional arterial compliance measures in predicting working memory performance measured by the OSPAN task. This cumulative evidence supports the utility of optical imaging as a complementary method for assessing the status of the brain’s arterial system and its consequences on cognition function and brain anatomy, in conjunction with other traditional methods such as MRI and Doppler ultrasound.

## Supporting information

S1 Data (XLSX)
